# Zinc Aspartate Induces IL-16 Secretion and Apoptosis in Human T Cells

**DOI:** 10.3390/biomedicines9030246

**Published:** 2021-03-01

**Authors:** Dirk Reinhold, Karina Guttek, Annika Reddig, Linda Voss, Claudia Schubert, Sascha Kahlfuss, Kurt Grüngreiff, Burkhart Schraven, Annegret Reinhold

**Affiliations:** 1 Institute of Molecular and Clinical Immunology, Medical Faculty, Otto-von-Guericke-University Magdeburg, 39120 Magdeburg, Germany; dirk.reinhold@med.ovgu.de (D.R.); karina.guttek@med.ovgu.de (K.G.); annika.reddig@med.ovgu.de (A.R.); linda.voss@med.ovgu.de (L.V.); clschube@gmail.com (C.S.); sascha.kahlfuss@med.ovgu.de (S.K.); burkhart.schraven@med.ovgu.de (B.S.); 2 Health Campus Immunology, Infection and Inflammation (GC-I3), Medical Faculty, Otto-von-Guericke-University Magdeburg, 39120 Magdeburg, Germany; 3 Klinikum Altenburger Land GmbH, Clinic of Gastroenterology/Hepatology, 04600 Altenburg, Germany; 4 Institute of Medical Microbiology and Hospital Hygiene, Infection Immunology, Medical Faculty, Otto-von-Guericke-University Magdeburg, 39120 Magdeburg, Germany; 5 Clinic of Gastroenterology, City Hospital Magdeburg GmbH, Birkenallee 34, 39130 Magdeburg, Germany; dr.kgruengreiff@t-online.de

**Keywords:** zinc, T cells, proliferation, cytokine, IL-16, apoptosis

## Abstract

T cell activation mediates immunity to pathogens. On the flipside, T cells are also involved in pathological immune responses during chronic autoimmune diseases. We recently reported that zinc aspartate, a registered drug with high bioavailability, dose-dependently inhibits T cell activation and Th1/Th2/Th17 cytokine production of stimulated human and mouse T cells. To understand the suppressive effect of zinc on T cell function, we here investigated the influence of zinc aspartate on human T cells focusing on the secretion of immunosuppressive cytokines, induction of apoptosis, and caspase 3/7 activity. To this end, we monitored either freshly stimulated or pre-activated human T cells in the presence of zinc aspartate from 40–140 µM over a period of 72 h. Under both experimental conditions, we observed a dose-dependent suppression of human T cell proliferation. While IL-1ra, latent TGF-β1, and IL-10 were dose-dependently reduced, we, unexpectedly, detected elevated levels of IL-16 upon zinc supplementation. In addition, the number of cells with active caspase 3/7 and, consecutively, the amount of cells undergoing apoptosis, steadily increased at zinc aspartate concentrations exceeding 100 µM. Taken together, our findings suggest that zinc aspartate impairs T cell fitness and might be beneficial for the treatment of T cell-mediated autoimmune diseases.

## 1. Introduction

Zinc is one of the most abundant essential trace elements in the human body. It acts as a cofactor of numerous enzymes. Zinc homeostasis is controlled by zinc binding proteins and zinc transporters [1]. Within the immune system, a wide range of cellular functions such as proliferation, differentiation, and survival are regulated by zinc [2].

An impairment of the precisely controlled zinc levels affects both the innate and adaptive immune system [3]. Zinc deficiency can result in reduced chemotaxis, oxidative burst and phagocytosis of neutrophils [4]. In addition, natural killer (NK) cells show reduced lytic activity [5]. Monocytes reveal impaired production of proinflammatory cytokines in the presence of zinc [6]. Within the adaptive immune system, zinc deficiency can lead to attenuated B cell function, decreased T cell proliferation, and impaired Th1/Th2 polarization [7,8]. Based on this knowledge, therapeutic zinc supplementation was suggested to reduce increased susceptibility to infections, a clinical manifestation of zinc deficiency. A meta-analysis of six randomized double-blind placebo-controlled clinical studies was conducted to elucidate the effect of adjunct zinc therapy in patients with severe pneumonia. In these studies, zinc significantly reduced the mortality but no significant effects on treatment failure and change of antibiotic therapy were observed [9].

Autoimmunity might be another manifestation of zinc deficiency. Sanna et al. reported significantly lower zinc concentrations in patients with multiple sclerosis (MS), type 1 diabetes or rheumatoid arthritis (RA) compared to healthy control individuals [10]. Zinc supplementation in MS patients showed no improvements of neurological symptoms compared with placebo [11]. As reviewed by Overbeck et al., most studies investigating zinc treatment in patients with RA also showed no beneficial effect [12]. Whereas the association between decreased serum zinc and autoimmune diseases was considered in systematic meta-analysis studies, the results of zinc therapy in patients with MS and RA are so far inconsistent and conflicting.

The beneficial effect of therapeutic zinc supplementation was convincingly demonstrated in experimental autoimmune encephalomyelitis (EAE), the accepted animal model of multiple sclerosis. Zinc aspartate reduced the severity of EAE in SJL mice after intraperitoneal and even after oral application [13,14,15]. For this model, it is well established that encephalitogenic Th1 and Th17 cells are the main effector cells of central nervous system (CNS) infiltration, demyelination, and axonal loss [16]. Kitabayashi et al. provided evidence that zinc inhibits the development of Th17 cells so that a milder EAE disease course was observed [17]. In addition to the downregulation of Th17 cells, zinc-mediated upregulation of regulatory T cells was reported [18].

These results of EAE experiments were supported by in vitro data showing that zinc suppressed the proliferation of freshly stimulated mouse and human T cells and reduced the production of Th1 and Th17 cytokines [13,15]. Moreover, the proliferation and cytokine production of pre-activated human T cells (T cell blasts) were inhibited by zinc aspartate [19]. In order to clarify the mechanism underlying this anti-proliferative effect, here, we investigated the influence of zinc aspartate on secretion of immunosuppressive cytokines, apoptosis rate, and caspase 3/7 activity of human T cells.

## 2. Materials and Methods

### 2.1. Reagents

Zinc aspartate (Unizink^®^), a registered pharmaceutical infusion solution with good bioavailability, was purchased from Köhler Pharma GmbH (Alsbach-Hähnlein, Germany). For human T cell stimulation, hybridoma supernatants of mouse anti-human CD3ε (OKT-3) and CD28 (248.23.2) monoclonal antibodies were produced in our lab.

### 2.2. Cells

Human peripheral blood mononuclear cells (PBMC) were isolated by Ficoll gradient (Biochrom, Berlin, Germany) centrifugation of heparinized blood collected from healthy volunteers. Human T cells were enriched by non-T cell depletion using the “Pan T cell isolation kit II” (Miltenyi Biotec GmbH, Bergisch Gladbach, Germany). The purity of isolated pan T cells in all experiments was routinely >97%. Isolated cells were washed twice and re-suspended in serum-free AIM-V culture medium (Invitrogen, Eggenstein, Germany). The study was reviewed and approved by the Ethics committee of the Medical faculty of the Otto-von-Guericke-University Magdeburg (No. 141/19, date of approval 17/9/2019) and all donors provided their written informed consent to participate in the study.

### 2.3. Proliferation, Cytotoxicity, and Apoptosis Assays Using the IncuCyte S3 Live-Cell Imaging System

Resting human T cells (3 × 10^4^ cells/100 µL) were incubated in quadruplicate cultures in 96-well microtiter culture plates (TPP Techno Plastic Products AG, Trasadingen, Swizerland) coated with anti-CD3ɛ (OKT-3) and anti-CD28 (248.23.2) monoclonal antibodies. To investigate the effect on freshly stimulated T cells, increasing concentrations of zinc aspartate or, as a control, vehicle controls were added at the beginning of the indicated experiments. To study the effect on pre-activated T cells, zinc aspartate or vehicle controls were added 48 h after initial stimulation.

For fluorescence-based cytotoxic as well as cell death analysis, 1.6 µM IncuCyte™ Caspase 3/7 Reagent and 250 nM IncuCyte™ CytoTox Red Reagent (both Essen Bioscience, Ann Arbor, MI, USA) were added. The IncuCyte S3 Live-Cell Imaging system (Essen Bioscience) was used for kinetic monitoring of cell proliferation, cytotoxicity, and caspase 3/7 activity in T cells. Every 3 h, images were taken of each well at 10× magnification for the duration of 72 h. Images of phase contrast mode as well as green and red fluorescence were monitored and automatically quantified by the IncuCyte S3 v2018B software (Essen Bioscience). Images were analyzed for numbers of green objects and red objects per well. T cell proliferation was presented as cell cluster area per well (µm^2^/well). The minimal area of a proliferation cell cluster (8–10 activated T cells) has been defined as >1200 µm^2^.

### 2.4. Determination of Cytokine Concentration by Enzyme-Linked Immunosorbent Assay (ELISA)

For measurement of cytokine secretion, human T cells (10^6^ cells/mL) were cultured at 37 °C in 24-well plates in AIM-V medium stimulated with plate-bound anti-CD3 and anti-CD28 antibodies. To investigate the effect on freshly stimulated T cells, increasing concentrations of zinc aspartate or vehicle controls were added at the beginning of each experiment. To study the effect on pre-activated T cells, zinc aspartate or vehicle controls were added 48 h after stimulation. Supernatants were harvested after 72 h of incubation and cytokine concentrations (IL-1 receptor antagonist (IL-1ra), latent TGF-β1, IL-10 and IL-16) were determined using specific ELISA kits (bio-techne Ltd., Minneapolis, MN, USA) according to manufacturer’s instructions. To release latent TGF-β1, samples were tested after transient acidification [20].

### 2.5. Apoptosis Measurements

T cell apoptosis was measured using an Annexin V-FITC Apoptosis Detection Kit with propidium iodide (PI, BioLegend, San Diego, CA, USA). Briefly, cells were washed with PBS and re-suspended in 1x binding buffer. Thereafter, cells (0.5 × 10^6^/100 µL) were incubated with 2.5 µL of Annexin V-FITC and 2.5 µL of PI for 15 min at room temperature in the dark, terminated by addition of 200 µL of 1 × binding buffer. Samples were analyzed by flow cytometry using LSRFortessa (BD Biosciences, Franklin Lakes, NJ, USA). Early apoptotic cells were defined by Annexin V positive and PI negative staining. Late apoptotic and non-viable cells were identified based on Annexin V and PI double positive staining. At least 20,000 cells were examined for each sample. Data was analyzed using the FlowJo software (version 7.6.4, Treestar Inc., Ashland, OR, USA).

### 2.6. Statistical Analysis

Statistical significances of proliferation, cytokine concentrations, and apoptosis measurements were calculated by repeated measures of One-Way ANOVA and Dunnett’s Multiple Comparison Analysis Test as post hoc test using GraphPad Prism software (version 8.0). For statistical analysis of proliferating cell clusters, CytoTox Red positive cells, and Caspase 3/7 positive cells, the respective measured values at the endpoint of 72 h were compared. *p*-values are indicated as follows: *p* < 0.05 (*); *p* < 0.01 (**); *p* < 0.001 (***).

## 3. Results

### 3.1. Zinc Aspartate Dose-Dependently Suppresses the Formation of Proliferating Cell Clusters

**Figure 1:**
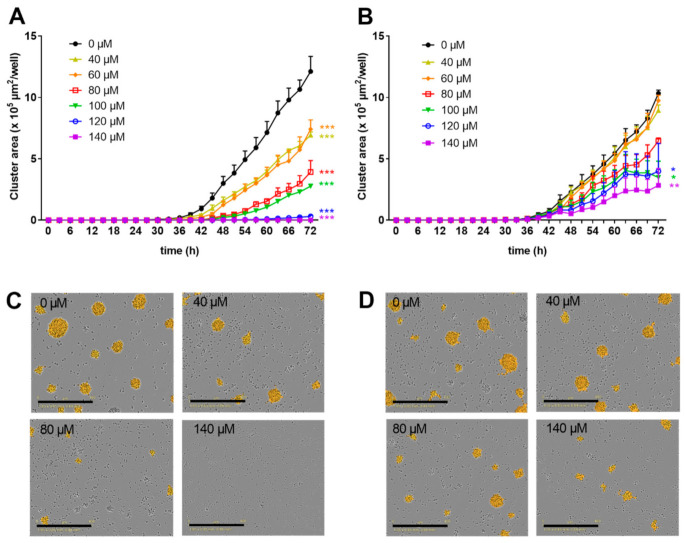
Zinc aspartate suppressed proliferation in freshly stimulated and pre-activated T cells. Human resting T cells were freshly stimulated (**A**) or pre-activated for 48 h (**B**) with anti-CD3/CD28 antibodies and cultured with increasing concentrations of zinc aspartate. Kinetic measures of the cluster area per well were recorded by the IncuCyte S3 imaging system at 3 h intervals for 72 h. Data are presented as the mean + standard error of the mean (SEM) from three independent experiments. (* *p* < 0.05, ** *p* < 0.01, *** *p* < 0.001). Representative images of proliferating cell clusters from freshly stimulated (**C**) or pre-activated (**D**) T cells treated with the indicated concentration of zinc aspartate are shown. Scale bar, 400 µM.

We previously demonstrated that zinc aspartate suppresses the proliferation of freshly stimulated human T cells [13,15] as well as of pre-activated human T cell blasts [19] in vitro. In order to investigate the dose-dependency of this inhibition in more detail, we here monitored the formation of proliferating cell clusters every 3 h over a period of 3 days using an IncuCyte S3 Live-cell imaging system. Proliferation of freshly isolated human T cells stimulated with anti-CD3 and anti-CD28 antibodies was partially inhibited by zinc aspartate at concentrations of 40–100 µM. Of note, 120 and 140 µM zinc aspartate completely blocked the formation of proliferating cell clusters (Figure 1A).

Next, resting human T cells were pre-activated with anti-CD3 and anti-CD28 antibodies for 48 h. Subsequently, increasing concentrations of zinc aspartate were added to the T cell blasts. Low zinc concentrations of 40 and 60 µM did not affect proliferation of T cell blasts compared to untreated controls. However, 80–140 µM zinc aspartate dose-dependently inhibited proliferation. Of note, even the highest concentration of zinc aspartate used did not completely block the formation of proliferating cell clusters of pre-activated T cells (Figure 1B). In line with these findings, microscopic images confirmed the observed strong negative correlation between the number of proliferating cell clusters and the concentration of zinc aspartate (Figure 1C,D).

### 3.2. Zinc Aspartate Inhibits Production of Anti-Inflammatory Cytokines, but Induces IL-16 Secretion

**Figure 2:**
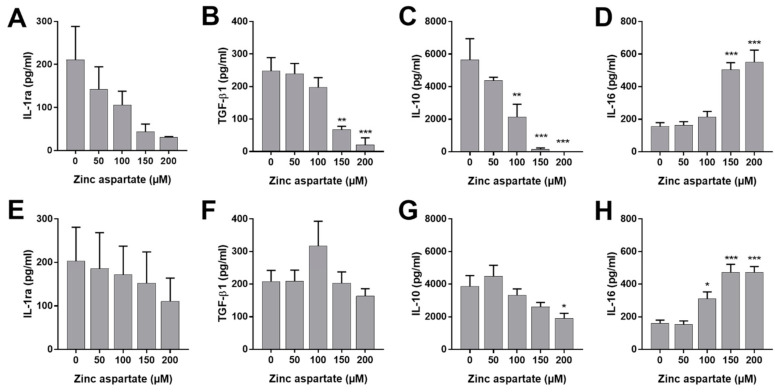
Zinc aspartate enhanced secretion of IL-16 from stimulated human T cells. Human T cells freshly stimulated (**A**–**D**) or pre-activated (**E**–**H**) with anti-CD3/CD28 antibodies were incubated in the presence or absence of different concentrations of zinc aspartate. Cell culture supernatants were harvested after 72 h of incubation. Cytokine concentrations were determined with specific ELISA. Data are given as mean + SEM of four independent experiments. (* *p* < 0.05, ** *p* < 0.01, *** *p* < 0.001).

To rule out the possibility that the observed anti-proliferative effect of zinc was the consequence of the production of immunosuppressive cytokines by T cells, we measured cytokine concentrations in the supernatants of freshly stimulated and pre-activated T cells following incubation with zinc aspartate. In this context, IL-1ra, latent TGF-β1, and IL-10 are best known for their immunosuppressive effects [21,22]. In addition, we measured the multifunctional cytokine IL-16 [23,24]. As shown in Figure 2, 150 and 200 µM of zinc aspartate significantly reduced the production of IL-1ra, latent TGF-β1, and IL-10 in freshly stimulated human T cells (Figure 2A–C). In contrast, this effect was completely absent in pre-activated T cells, with the exception of IL-10 inhibition at 200 µM zinc aspartate (Figure 2E–G). Interestingly, incubation of stimulated T cells with high concentrations of zinc aspartate significantly enhanced the secretion of IL-16. Increased IL-16 release was observed in freshly stimulated as well as pre-activated human T cells (Figure 2D,H). Thus, our data show that zinc aspartate selectively induces IL-16 secretion of human T cells.

### 3.3. Zinc Aspartate Induces T Cell Apoptosis at Concentrations Above 100 µM

**Figure 3:**
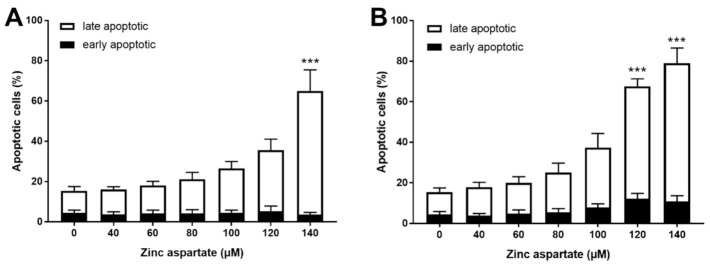
Zinc aspartate induced late apoptosis in freshly stimulated and pre-activated T cells. Resting human T cells were freshly stimulated (**A**) or pre-activated for 48 h (**B**) with anti-CD3/CD28 antibodies and cultured with increasing concentrations of zinc aspartate. Cells were stained after 72 h with AnnexinV-FITC/propidium iodide (PI) for flow cytometric analysis. Quantification of early and late apoptotic cells of *n* = 4 independent experiments are shown. Statistical analysis was performed with One-Way ANOVA and Dunnett’s Multiple Comparison Analysis Test as post hoc test. (*** *p* < 0.001).

IL-16 is considered a “dual-function cytokine.” It regulates CD4 T cell function and acts as a chemoattractant [23]. In addition, when released during late apoptosis and necrosis, IL-16 may function as alarmin [24]. We wondered whether enhanced release of IL-16 after treatment of stimulated T cells with zinc aspartate might be associated with the induction of apoptosis. Based on this consideration, we measured AnnexinV binding and PI staining to discriminate between early (AnnexinV+PI-) and late apoptosis (AnnexinV+PI+). Zinc aspartate concentrations above 100 µM significantly increased the percentages of late apoptotic T cells 72 h after stimulation. This effect was even more pronounced in pre-activated T cells compared to freshly stimulated T cells (Figure 3). Of note, we did not observe any effect of zinc aspartate on T cell apoptosis at concentrations below 100 µM. In contrast to the increased late apoptosis, the percentage of AnnexinV single positive early apoptotic cells remained unchanged. Taken together, we show here that concentrations of zinc aspartate of 120 and 140 µM increased the percentage of AnnexinV/PI double positive cells. We thus conclude that zinc aspartate induced apoptosis in stimulated T cells at concentrations above 100 µM.

### 3.4. Zinc Aspartate Induces Loss of Membrane Integrity at Concentrations above 80 µM

**Figure 4:**
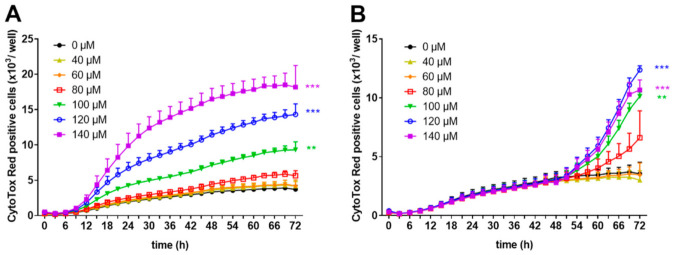
Zinc aspartate impaired membrane integrity of freshly stimulated and pre-activated T cells. Human resting T cells were freshly stimulated (**A**) or pre-activated for 48 h (**B**) with anti-CD3/CD28 antibodies and cultured with increasing concentrations of zinc aspartate. For kinetic analyses, T cells were cultured with increasing concentrations of zinc aspartate in the presence of CytoTox Red reagent. Kinetic measures of the number of CytoTox Red positive cells were recorded by the IncuCyte S3 imaging system at 3 h intervals for 72 h. Data are presented as the mean + SEM from three independent experiments (** *p* < 0.01, *** *p* < 0.001).

To prove the hypothesis that high concentrations of zinc aspartate induce apoptosis, we investigated the effect of zinc aspartate under the two experimental conditions in the presence of the IncuCyte™ CytoTox Red Reagent using the IncuCyte S3 Live-cell imaging system. This highly sensitive cyanine nucleic acid dye only penetrates cells with disturbed membrane integrity. Zinc aspartate at concentrations of 40, 60, and 80 µM did not show any cytotoxic effects on freshly stimulated human T cells. In contrast, increasing concentrations of zinc aspartate from 100–140 µM significantly enhanced loss of T cell membrane integrity compared to untreated control cells (Figure 4A).

Membrane integrity of 48 h pre-activated human T cell blasts was unaffected by the addition of zinc aspartate at concentrations of 40 and 60 µM and showed the same curve progression as untreated cells. Higher concentration ranging from 80–140 µM zinc aspartate also increased the number of CytoTox Red positive T cell blasts (Figure 4B). However, the effect on pre-activated human T cells was less intense compared to the effect on freshly stimulated T cells (10,669 CytoTox Red positive cells/well vs.18,185 CytoTox Red positive cells/well at 140 µM zinc aspartate after 72 h; Figure 4A, B). These results indicate that high concentrations of zinc aspartate (above 80 µM) inhibit proliferation of anti-CD3/CD28 antibody-stimulated human T cells and induce loss of membrane integrity.

### 3.5. Zinc Aspartate Increases Activity of Caspase 3 and 7 at Concentrations above 100 µM

**Figure 5:**
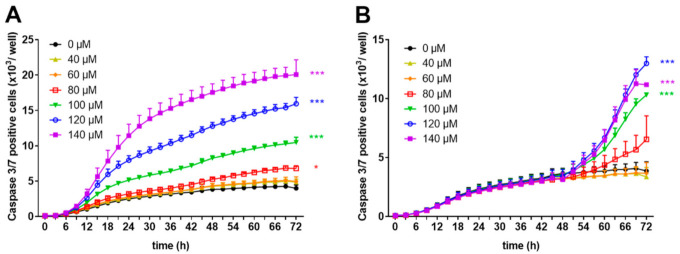
Zinc aspartate activated caspase-3 and caspase 7 in freshly stimulated and pre-activated T cells. Human resting T cells were freshly stimulated (**A**) or pre-activated for 48 h (**B**) with anti-CD3/CD28 antibodies and cultured with increasing concentrations of zinc aspartate. For kinetic analyses, T cells were cultured with increasing concentrations of zinc aspartate in the presence of Caspase-3/7 reagent. Kinetic measures of the number of caspase 3/7 positive cells were recorded by the IncuCyte S3 imaging system at 3 h intervals for 72 h. Data are presented as the mean + SEM from three independent experiments. (* *p* < 0.05, *** *p* < 0.001).

Loss of membrane integrity is a typical hallmark of late apoptosis/necrosis [25]. To perform real-time evaluation of cell apoptosis, we used a proteolytic substrate (DEVD) which is cleaved by active effector caspases 3 and 7, resulting in fluorescent staining of nuclear DNA. Using this reagent, we monitored the number of caspase 3/7 positive cells within freshly stimulated or pre-activated human T cells in the presence or absence of zinc aspartate using the IncuCyte S3 Live-cell imaging system. As shown in Figure 5A, the release of the cleaved fluorescent substrate steadily increased in cells incubated with zinc concentrations in the range of 100–140 µM. Lower zinc aspartate concentrations (40–80 µM) did not change the number of caspase 3/7 positive cells.

Incubating pre-activated T cells with high concentrations of zinc aspartate (100–140 µM) resulted in caspase 3/7 activation, whereas incubation with lower concentrations of zinc aspartate (40–60 µM) showed no effect or only a slight increase (80 µM zinc aspartate) in caspase 3/7 positive cells (Figure 5B). Taken together, high concentrations of zinc aspartate induced an increased amount of cells with disturbed membrane integrity in parallel with elevated number of cells expressing active caspase 3/7, indicating the induction of apoptosis in both freshly stimulated and pre-activated human T cells.

## 4. Discussion

The trace element zinc plays a regulatory role in the immune system by influencing cells and humoral factors of both the innate and adaptive immune system. In the past, several research groups investigated the influence of different zinc compounds on T cell activation and proliferation in vitro and in vivo.

Our group previously reported that zinc aspartate significantly inhibits the proliferation of human T cells after stimulation with anti-CD3/CD28 antibodies. These experiments were performed using the incorporation of 3H-thymidine as endpoint measurement [13,15,19]. In the present study, we applied an IncuCyte S3 Live-cell imaging system to investigate the influence of zinc aspartate on T cell proliferation. As pre-activated autoreactive T cells are the main effectors in the immunopathogenesis of several autoimmune diseases, we studied the effect of zinc aspartate not only on freshly stimulated T cells but also on pre-activated T cell blasts. We confirmed that zinc aspartate suppresses the proliferation of freshly stimulated as well as of pre-activated human T cells. Using the kinetic measurement of the number of proliferating cell clusters every three hours over a period of three days, we could demonstrate the inhibitory effect of zinc on proliferation already at 48 h for freshly stimulated T cells and at 60 h (12 h after addition of zinc aspartate) for pre-activated T cells.

Furthermore, zinc aspartate significantly inhibits the production of Th1 (IL-2, IFN-γ, TNF-α), Th2 (IL-5), and Th17 cytokines (IL-17, GM-CSF) of freshly stimulated T cells in a dose-dependent manner [13,15]. Moreover, we previously found that zinc aspartate has the specific capacity to suppress the proliferation and Th1/Th2/Th17 cytokine production of pre-activated human T cell blasts in vitro [19]. In the present study, we now provide evidence that zinc aspartate dose-dependently reduces the production of the immunosuppressive cytokines IL-1ra, IL-10, TGF-β1.

Several groups have previously described that zinc is involved in regulation of T cell activation, differentiation, and cytokine-mediated proliferation [26]. In this context, zinc was also shown to alter the IL-6/STAT3 signalling cascade and, thereby, to inhibit Th17 development [17]. On the other side, zinc enhances TGF-β1 signalling in T cells and increases the number of regulatory T cells [18]. By showing that different immunosuppressive cytokines, such as latent TGF-β1 and IL-10, are inhibited by zinc, we could rule out the possibility that an increased production of these immunosuppressive cytokines is the reason for impaired T cell function in the presence of zinc.

Unexpectedly, we found under both conditions of stimulation that zinc aspartate at pharmacological concentrations above 100 µM induced the secretion of the multifunctional cytokine IL-16. To the best of our knowledge, this is the first report describing release of IL-16 by human T cells after zinc treatment. T lymphocytes are one of the major sources of IL-16. Following T cell activation, the precursor molecule pro IL-16 is cleaved by caspase 3 and mature IL-16 is released. This bioactive IL-16 can form homotetramers that bind to the CD4 co-receptor and regulate function of CD4+ T cells. IL-16 controls migration, activation, proliferation, and interaction of CD4+ T cells with antigen-presenting cells (APC) [23]. In addition to this immunoregulatory role, recent reports described IL-16 as an alarmin, which is a protein released by dying cells that thereby mediates stress signals that are sensed by other cells [24]. Considering the latter two biological functions, IL-16 was termed a “dual-function cytokine”.

We asked whether enhanced IL-16 secretion after incubation of activated T cells with zinc aspartate might be a consequence of apoptosis. Indeed, we observed zinc-mediated loss of membrane integrity and an increase in caspase 3/7 activity in our T cell systems. Application of specific dyes and measurements using the IncuCyte S3 Live-cell imaging system allowed kinetic monitoring over 72 h. The kinetic data were confirmed by independent end-point measurements of AnnexinV/PI staining using flow cytometry. Thus, we interpret the increased release of IL-16 as a consequence of disturbed cell membrane integrity and late apoptosis. In addition, the released IL-16 might further suppress T cell proliferation. Cruikshank et al. showed that preincubation of T cells with recombinant IL-16 inhibited T cell activation and proliferation. This effect was demonstrated for anti-CD3 stimulation [27] as well as for mixed lymphocyte reaction (MLC) of human T cells [28]. Furthermore, it was shown that IL-16 stimulation can induce the expansion of FoxP3+ regulatory T cells. This observation provides a potential mechanism of the immunosuppressive effect of IL-16 [29].

Twenty-five years ago, the possible effect of zinc on apoptosis was reviewed by Sunderman [30]. However, the data were conflicting. Dependent on the concentration and the experimental conditions, zinc supplementation either protected against apoptosis or did not. Later, it was shown that zinc can induce apoptosis in human peripheral blood mononuclear cells (PBMC) [30]. Chang et al. differentiated between physiological (2–15 µM) and pharmacological (15–100 µM) zinc concentration. The authors demonstrated that incubation of resting PBMC with zinc sulfate at concentrations of 100 µM or higher resulted in induction of apoptosis and activation of the caspase 3 pathway [31]. In agreement with these results, our study shows impaired membrane integrity and enhanced membrane permeability and increased percentage of late apoptotic cells at 48 and 72 h of incubation with ≥100 µM zinc aspartate in freshly stimulated as well as in pre-activated human T cells. Another study performed by Ostan et al. provided evidence that zinc sulfate at physiological concentrations of 12.5 µM can increase oxidative stress-induced apoptosis in PBMC. This effect was observed in old subjects and nonagenarians but was absent in cells from young donors [32].

Thus, it is now clear that zinc can induce apoptosis in stimulated T cells at pharmacological concentrations of 100 µM or higher. In line with this observation, it should be noted that excessive zinc might cause unwanted inflammatory reactions via the release of IL-16. In contrast, the lowest concentration of 40 µM zinc aspartate used in our experimental system (near to physiological zinc levels) did not induce IL-16 release and had no effect on apoptosis induction. Therefore, pharmacological zinc supplementation has to be carefully monitored, especially in elderly patients.

In the present study, we deepened our understanding how zinc suppresses T cell activation. Our data show induction of apoptosis in zinc-treated T cells. This effect was accompanied by enhanced secretion of the multifunctional cytokine IL-16 in vitro. We investigated here the proliferative response, IL-16 secretion, and apoptosis induction of the pan T cell population. It remains to be determined whether the CD4+ and CD8+ subpopulations of T cells react differently in response to zinc aspartate. Given the fact that IL-16 can directly interact with the CD4 receptor, a more pronounced effect of zinc on CD4+ T cells is suggested. Further investigations will focus on this question. Taken together, our data provide evidence that at defined concentrations, zinc aspartate induces apoptosis and exerts an anti-proliferative effect on activated human T cells. For a long time, therapeutic zinc supplementation has been discussed as therapy for the treatment of T cell-mediated autoimmune diseases. Further studies should be performed to prove this option of controlled immunosuppressive zinc therapies for such autoimmune diseases.

## Data Availability

Data is contained within the article.
